# Validierung des „Total Morbidity Scores“ und Untersuchung der Wirksamkeit von Methotrexat bei der lokalisierten Sklerodermie

**DOI:** 10.1007/s00393-022-01296-0

**Published:** 2022-12-15

**Authors:** Ann-Kathrin Hoppe, Suzanne C. Li, Ivan Foeldvari

**Affiliations:** 1 Semmelweis University, Üllői út 26, 1086 Budapest, Hungary; 2 Department of Pediatrics, Division of Pediatric Rheumatology, Hackensack Meridian School of Medicine, 07601 Hackensack, NJ USA; 3 Hamburger Zentrum für Kinder- und Jugendrheumatologie, Hamburg, Deutschland

**Keywords:** Autoimmunerkrankung, Kinderrheumatologie, Sklerose, Fibrose, Damage Index, Autoimmune disease, Pediatric rheumatology, Sclerosis, Fibrosis, Damage index

## Abstract

**Hintergrund:**

Die lokalisierte Sklerodermie ist eine Autoimmunerkrankung aus der Gruppe der Kollagenosen, welche sich kutan und extrakutan manifestieren kann. Die extrakutanen Manifestationen können eine signifikante Morbidität haben, werden aber in bisherigen Scoresystemen nicht berücksichtigt. Aus diesem Grund wurde ein weiteres Scoringsystem, der „Total Morbidity Score“ (TMS), entwickelt. Dieser berücksichtigt auch die extrakutanen Symptome.

**Methodik:**

Im Rahmen der retrospektiven monozentrischen Studie am Hamburger Zentrum für Kinder- und Jugendrheumatologie wurde der Total Morbidity Score bei Patienten von 2004 bis 2019, welche an lokalisierter Sklerodermie erkrankt sind, angewandt, die mindestens eine Kontrollvorstellung hatten. Zudem wurden die Daten nach den bisherigen etablierten Scoresystemen Localized Scleroderma Cutaneous Assessment Tool (LoSCAT) ausgewertet, um eine bessere Vergleichbarkeit zum TMS zu gewährleisten. Im Weiteren wurden die Scorewerte im Verlauf unter der Therapie mit Methotrexat betrachtet und verglichen.

**Ergebnisse:**

Aufgrund fehlender Kontrollvorstellungen konnten von den 95 Patienten mit gesicherter Diagnose Daten von 51 Patienten in die retrospektive Auswertung einfließen. Die Behandlung dieser Patienten wurde über einen Zeitraum von 2 Jahren betrachtet, von der Erstvorstellung über mindestsens 3 weitere Kontrollvorstellungen. Der TMS Gesamtscore blieb weitestgehend konstant. Es zeigt sich ein schwacher Zusammenhang zwischen TMS-Gesamtscore und Localized Scleroderma Skin Damage Index (mLoSDI), welcher den Grad der Schädigung anzeigt. Zudem konnten unwesentliche Veränderungen des TMS-Gesamtscores über die Zeit unter MTX-Therapie gezeigt werden (T1/T4: −0,007).

**Diskussion:**

Die Auswertung hat gezeigt, dass sich der TMS-Gesamtscore hauptsächlich aus den extrakutanen Manifestationen speist, was die Ungenauigkeit der bisherigen Scores aufzeigt. Ein weiterer Vorteil des TMS ist, dass verschiedene Punktwerte vergeben werden, je nachdem, ob das Merkmal neu auftritt, persistiert, sich verbessert oder gar verschlechtert. Der TMS ist zeitintensiver zu erheben, aber lässt eine genauere Beurteilung der Krankheitsaktivität zu.

## Abstract (en)

**Background:**

Localized scleroderma is an autoimmune disease belonging to the group of collagenoses, which can manifest cutaneously and extracutaneously. The extracutaneous manifestations may have significant morbidity but are not considered in previous scoring systems. For this reason, another scoring system, the total morbidity score (TMS) was developed, which also takes into account the extracutaneous symptoms.

**Method:**

In the retrospective monocentric study at the Hamburg Center for Pediatric and Adolescent Rheumatology, the TMS was applied to patients from 2004–2019 suffering from localized scleroderma who had at least one control presentation. In addition, data were analyzed according to the previously established localized scleroderma cutaneous assessment tool (LoSCAT) scoring systems to ensure better comparability to the TMS. Furthermore, the score values were considered and compared during the course of treatment with methotrexate (MTX).

**Results:**

Due to a lack of control presentations, data from 51 of the 95 patients with a confirmed diagnosis could be included in the retrospective evaluation. The treatment of these patients was considered over a period of 2 years, from the initial presentation over at least 3 further control presentations. The TMS total score remained largely constant. There was a weak correlation between the TMS total score and the localized scleroderma skin damage index (mLoSDI), which indicates the degree of damage. In addition, insignificant changes in the TMS total score were shown over time with MTX treatment (T1/T4: −0.007).

**Discussion:**

The evaluation showed that the TMS total score is mainly fed by the extracutaneous manifestations, demonstrating the inaccuracy of previous scores. Another advantage of the TMS is that different scores are assigned depending on whether the feature is new, persistent, improving, or even worsening. The TMS is more time consuming to collect but enables a more accurate assessment of disease activity.

Die lokalisierte Sklerodermie ist eine Autoimmunerkrankung, welche sich kutan und extrakutan manifestieren kann [1]. Insbesondere die extrakutanen Manifestationen erhöhen den Leidensdruck der Patienten. Diese werden in bisherigen Scoresystemen nicht berücksichtigt. Ziel der vorliegenden Publikation ist es, ein neues Scoringsystem anzuwenden, welches auch die extrakutanen Komponenten der Erkrankung beurteilt, der sog. „Total Morbidity Score“ (TMS). Der TMS evaluiert neben Hauterscheinungen auch Veränderungen am Muskel-Skelett-System, Größen- und Längendifferenz der Extremitäten, neurologische Beteiligungen und Veränderungen in anderen Organen, welche sich in 5 Module aufteilen und deren Werte addiert den TMS-Gesamtscore ergeben, um so eine bessere Beurteilung der Aktivität und Schwere der lokalisierten Sklerodermie sicherzustellen [2].

Bislang wird das Localized Scleroderma Cutaneous Assessment Tool (LoSCAT), welches den Localized Scleroderma Skin Severity Index (mLoSSI) und Localized Scleroderma Skin Damage Index (LoSDI) beinhaltet, zur Beurteilung der lokalisierten Sklerodermie verwendet. LoSDI und mLoSSI beziehen sich nur auf Erscheinungen und Läsionen im Bereich der Haut [4]. Zudem stellt Methotrexat nach wie vor das Mittel der Wahl in der Therapie der lokalisierten Sklerodermie dar [5]. Die Studienlage bestätigt einen positiven Effekt der MTX-Therapie auf die Aktivität der Erkrankungen [5]. Dessen Wirksamkeit auf extrakutane Symptome wurde zudem retrospektiv betrachtet.

## Methoden

Der TMS wurde im Rahmen einer retrospektiven monozentrischen Analyse auf die Patientendaten aus dem Hamburger Zentrum für Kinder- und Jugendrheumatologie mit gesicherter Diagnose juveniler lokalisierter Sklerodermie von 2004 bis 2019 angewandt. Die vorhandenen Patientendaten wurden zusätzlich nach mLoSSI und LoSDI ausgewertet, um eine Vergleichbarkeit zum TMS zu ermöglichen. In die Studie wurden Kinder eingeschlossen, die zum Zeitpunkt der Diagnosestellung unter 18 Jahre alt waren, eine konservative oder medikamentöse Therapie mit Methotrexat erhielten und eine entsprechende Compliance hinsichtlich Erscheinen zu Kontrollterminen und regelmäßiger Medikamenteneinnahme aufwiesen. Zum Ausschluss der Patienten kam es, wenn nur eine Initialvisite existierte. Im Rahmen der Studie wurden Daten zu Alter, Geschlecht, Zeitpunkt der Erstmanifestation, Zeitpunkt der Diagnosestellung, Therapiebeginn und Veränderungen des TMS, mLoSDI und mLoSSI unter Therapie erfasst. Der TMS-Wert wird aus 5 Modulen, Haut (maximal 3 Punkte) plus das prozentuale Ausmaß der Hautmerkmale im Verhältnis der Körperoberfläche (maximal 3 Punkte), muskuloskeletal (maximal 69 Punkte), Größen- und Längendifferenz (maximal 30 Punkte), Kopf/Neuro (maximal 73 Punkte) und andere Organbeteiligung (maximal 12 Punkte), gebildet, deren Werte addiert den TMS-Gesamtscore ergeben. Durch diesen ganzheitlichen Score lassen sich auch Therapieverläufe und -Erfolge besser beurteilen. Modul A bezieht sich auf Erscheinungen der Haut, wobei Dyspigmentierung, Atrophie der Haut und des subkutanen Gewebes und Hautverdickung betrachtet werden. Diese Veränderungen können sowohl in der Inspektion als auch sonographisch beurteilt werden. Die Punktwerte der einzelnen Erscheinungen werden nicht addiert, sondern es wird lediglich der höchste Wert angegeben. Modul B bewertet das muskuloskeletale System hinsichtlich Arthritis, Myositis, Fasziitis, Gelenkeinschränkungen im Bewegungsausmaß und Kontrakturen. Im Rahmen der Kontrollen werden die Bewegungsausmaße aller Gelenke inklusive der Wirbelsäule nach der Neutral-Null-Methode geprüft und Einschränkungen im Verhältnis zum physiologischen Bewegungsausmaß gesetzt. Je nach prozentualem Ausmaß werden unterschiedliche Punktwerte vergeben. Liegt der Verdacht einer Arthritis vor, werden diese Gelenke sonographisch beurteilt. Bei Vorliegen akuter Arthritis wird das Bewegungsausmaß nicht überprüft, sondern es werden lediglich verschiedene Punktwerte je nach Anzahl der betroffenen arthritischen Gelenke vergeben. Das nächste Modul bezieht sich auf Wachstumsdifferenzen von Extremitäten oder Rumpf, es werden Hemiatrophie an Rumpf, Brust und Gesäß und Umfangs- oder Längenunterschiede der Extremitäten beurteilt. Umfang und Länge der Extremitäten werden im Rahmen der Untersuchungen mittels Zentimetermaßband verglichen. Optisch ersichtliche Atrophien, insbesondere Fett- und Muskelatrophien, im Bereich der Brust, des Rumpfes und des Gesäßes werden sonographisch beurteilt und in mild oder mäßig schwer unterschieden. Im Anschluss folgt Modul D zur Beteiligung von Kopf und Nerven. Beurteilt werden Erkrankungen an Gehirn, Augen, Kiefer und Zähnen, Gesichtsverunstaltung durch Hautkontrakturen, Haarausfall und periphere Neuropathien. Eine regelmäßige, halbjährliche Mitbeurteilung des Augen- und Zahnarztes hinsichtlich inflammatorischer Erkrankungen, wie z. B. Uveitis, Konjunktivitis oder Keratitis, Visusänderungen, Augentrockenheit und Veränderungen des Kauapparates ist diesbezüglich obligat. Mildere neurologische Auffälligkeiten wie Kopfschmerzen können anamnestisch erhoben werden. EEG-Abnormalitäten, Krampfanfälle, Neuropathien, Myotonien und vaskuläre Abnormalitäten werden in diesem Modul ebenfalls erfasst. Das letzte Modul bezieht sich auf Erkrankungen anderer Organe. Hier werden Lunge, Gefäßsystem und Gastrointestinaltrakt beurteilt hinsichtlich Krankheitsbildern, die mit der lokalisierten Sklerodermie einhergehen können, aber seltenere Probleme darstellen, wie beispielsweise Raynaud-Phänomen, vaskulitischer Ausschlag, verminderte Lungenfunktion, Dyspnoe, Obstipation, Diarrhö oder GERD. In den letzten beiden Modulen werden unterschiedliche Punktwerte verteilt, je nachdem, ob die Erscheinung neu, verbessert, stabil oder verschlechtert ist. Somit ergibt sich mit dem TMS ein Maß, das die Bewertung des gesamten Spektrums der LS-Morbidität und die Entwicklung der extrakutanen Manifestationen im Verlauf ermöglicht [2]. T1 bezeichnet den Zeitpunkt der Erstvorstellung und den Start der Therapie. Die weiteren Auswertungen erfolgten im Abstand von 6 Monaten. Die statistische Auswertung der Daten erfolgte durch deskriptive Methoden, dem gemischten Poisson-Modell, gemischter negativer Binominalverteilungsmodelle und der R Core Team-Software (R Core Team. 2021. R: A Language and Environment for Statistical Computing. Vienna, Austria: R Foundation for Statistical Computing) [6].

MTX-Therapie wurde über die ersten 24 Monate der Therapie ausgewertet von der Erstvorstellung über mindestens 3 weitere Kontrollvorstellungen.

Ein Ethikantrag bei retrospektiven Auswertungen von pseudonymisierten Daten bei Einverständnis der Patienten/Eltern wird von der Hamburger Ethikkommission nicht benötigt.

## Ergebnisse

Aufgrund der Ausschlusskriterien konnten von den 95 Patienten mit gesicherter Diagnose Daten von 51 Patienten in die retrospektive Auswertung einfließen.

**Figure 1:**
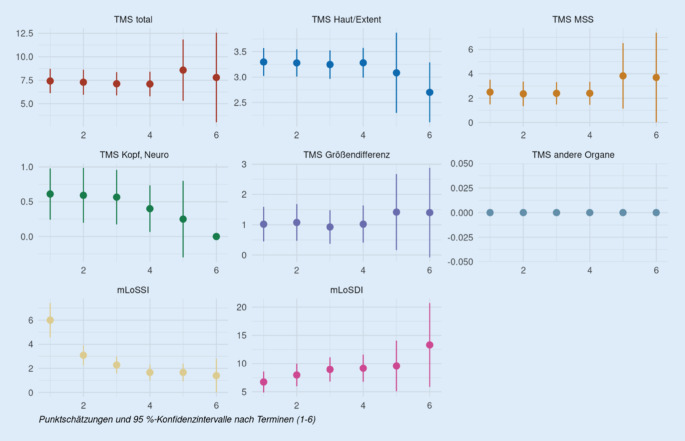

**Figure 2:**
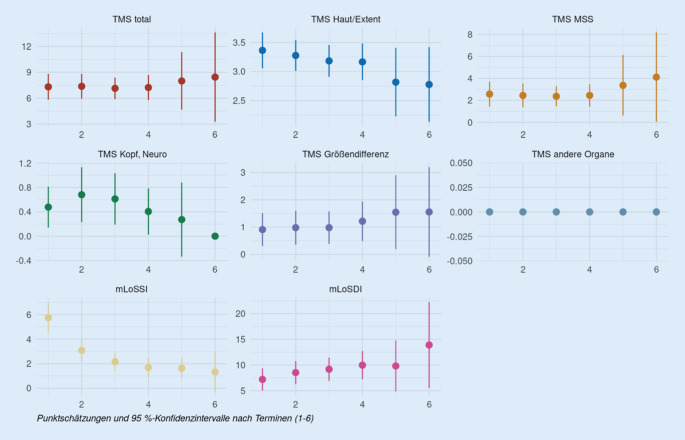

Der Total Morbidity Score wurde auf diese 51 Patientendaten angewandt und mit den aktuell verwendeten Scores, dem mLoSSI und mLoSDI, verglichen. Die Auswertung erfolgte über einen Zeitraum von 15 Jahren, von 2004 bis 2019. Die Behandlung der Patienten wurde über die ersten 24 Monate der Therapie ausgewertet, von der Erstvorstellung über mindestens 3 weitere Kontrollvorstellungen. Die Abb. 1 und 2 zeigen den Verlauf der einzelnen TMS-Unterpunkte und der LoSCAT-Werte. Es wurden einmal alle Patientendaten und in Abb. 2 lediglich die MTX-Patienten betrachtet.

Der TMS Gesamtscore bleibt weitestgehend konstant (T1/T4: −0,1; T1/T6: +1,1). Bei der Größendifferenz gibt es eine marginale Steigerung (+0,3 bzw. +0,6), die Scores für Hauterscheinungen und Kopf-, neurologische Symptome weisen eine schwache Senkung auf (−0,2, −0,6 bzw. −0,07, −0,5). Der Score für muskuloskeletale Erscheinungen bleibt konstant bis T4 und weist danach eine Steigerung auf (T1/T6: +1,5). Die Werte des mLoSSI sinken im Verlauf (T1/T4: −4,0) und die Werte des mLoSDI steigen (T1/T4: +2,8).

**Figure 3:**
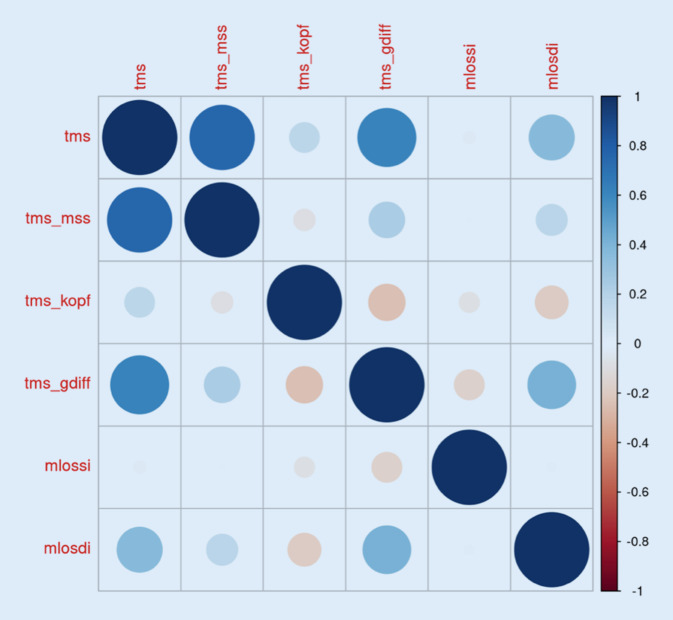

Die Abb. 3 zeigt die Korrelationen zwischen den TMS-Modulen, mLoSSI und mLoSDI. Es zeigt sich ein schwacher Zusammenhang zwischen TMS-Gesamtscore und mLoSDI. Zwischen den beiden LoSCAT-Werten besteht kein Zusammenhang (Abb. 3). Die Tab. 1 und 2 beziehen sich auf die Wirksamkeit von MTX. Die Tab. 1 zeigt unwesentliche Veränderungen des TMS-Gesamtscores über die Zeit unter MTX-Therapie (T1/T4: −0,007). Die Tab. 2 zeigt, dass die Werte des mLoSSI unter MTX-Therapie stark über die 6 Termine fallen. Beim 2. Termin, 6 Monate nach Erstvorstellung, liegt der Wert nur mehr etwas über 50 % des Basiswertes (0,526), nach dem 4. Termin, 24 Monate nach Erstvorstellung, gar nur noch bei rund 30 %. Somit zeigt sich ein deutlicher Rückgang der Aktivität unter MTX-Therapie (Tab. 2). Die mLoSDI-Werte steigen unter MTX-Therapie über die 6 Termine an (T1/T6: +0,39), was eine Zunahme der Schädigung bestätigt (Tab. 3).

**Table 1:** 

Term	Schätzung	Standardabweichung	Statistische Daten	*p*-Wert
(Achsenabschnitt)	1,854	0,102	18,170	< 0,001
tpt2	−0,015	0,079	−0,194	0,846
tpt3	−0,036	0,079	−0,460	0,646
tpt4	−0,022	0,082	−0,270	0,787
tpt5	−0,005	0,131	−0,037	0,970
tpt6	0,054	0,140	0,386	0,699

*tpt2–6* Kontrolltermin 2–6

**Table 2:** 

Term	Schätzung	Standardabweichung	Df	Statistische Daten	*p*-Wert
(Achsenabschnitt)	1,620	0,123	146	13,177	< 0,001
tpt2	−0,642	0,134	146	−4,806	< 0,001
tpt3	−0,994	0,149	146	−6,688	< 0,001
tpt4	−1,259	0,172	146	−7,303	< 0,001
tpt5	−1,167	0,329	146	−3,550	< 0,001
tpt6	−1,299	0,394	146	−3,297	0,001

*tpt2–6* Kontrolltermin 2–6

**Table 3:** 

Term	Schätzung	Standardabweichung	Statistische Daten	*p*-Wert
(Achsenabschnitt)	1,532	0,173	8,840	< 0,001
tpt2	0,187	0,076	2,458	0,014
tpt3	0,224	0,075	3,000	0,003
tpt4	0,256	0,076	3,370	< 0,001
tpt5	0,263	0,124	2,114	0,035
tpt6	0,579	0,121	4,771	< 0,001

*tpt2–6* Kontrolltermin 2–6

## Diskussion

Die retrospektive Analyse der Patientendaten unter Erhebung des Total Morbidity Scores zeigt, dass sich die Manifestation der lokalen Sklerodermie nicht nur auf die Hauterscheinungen bezieht, welche bislang als Einziges zur Beurteilung der Krankheitsaktivität und des Krankheitsverlaufs betrachtet wurden (Abb. 3). Wie auch schon weitere prospektive Studien zeigten, treten extrakutane Erscheinungen bei 40–70 % der lokalen Sklerodermie-Patienten auf [5, 7]. Der TMS-Gesamtscore speist sich hauptsächlich aus den Werten der muskuloskeletalen Erscheinungen und der Größen- und Längendifferenz der Extremitäten (Abb. 3). Diese Werte werden weder im mLoSSI noch im mLoSDI berücksichtigt. Ein weiterer Vorteil des TMS ist, dass verschiedene Punktwerte vergeben werden, je nachdem, ob das Merkmal neu auftritt, persistiert, sich verbessert oder gar verschlechtert. Dies lässt eine deutlich bessere Beurteilung der Krankheitsaktivität zu. Im mLoSSI gibt es lediglich zusätzliche Punkte für neu erschienene Hautveränderungen und Änderungen der Aktivität. Ebenso wie der LoSCAT ist der TMS leicht zu erlernen, einfach in der Anwendung, aber zeitintensiver.

Ein weiterer Aspekt der retrospektiven Analyse war, die Wirksamkeit von Methotrexat als Therapeutikum der juvenilen lokalisierten Sklerodermie zu zeigen. Das Ziel der MTX-Therapie ist es, eine Progression der Erkrankung zu verhindern und eine Remission zu erreichen [3]. Dieses Ziel lässt sich unter Verwendung des TMS wesentlich besser beurteilen als mit dem mLoSSI, der die Aktivität lediglich auf Basis der Hauterscheinungen belegt. Sowohl der mLoSSI als auch der TMS-Wert für die Hauterscheinungen fallen unter MTX-Therapie deutlich ab (Abb. 2). Der TMS-Gesamtscore bleibt unter MTX-Therapie allerdings recht konstant, was dem gewünschten Effekt entspricht. Berücksichtigt man lediglich den mLoSSI, würde man von keiner Krankheitsaktivität mehr sprechen. Der TMS mit seinen unterschiedlichen Modulen zeigt, dass die Krankheitsaktivität fällt, aber da der TMS Aktivität und Schädigung reflektiert, kann man kaum den Wert „0“ erreichen. Somit lässt sich eine deutlich bessere Beurteilung des Therapieverlaufs und Einstellung der Therapie vornehmen. Insgesamt liefert diese Analyse weitere Belege dafür, dass der TMS zusammen mit der Gesamtbeurteilung durch den Arzt ein angemessenes und fortschrittlicheres Maß für die Beurteilung der Behandlung von lokalisierter Sklerodermie bei Kindern ist.

## Fazit für die Praxis

- Extrakutane Erscheinungen treten bei 40–70 % der lokalisierten Sklerodermie-Patienten auf.
- Der TMS ist leicht zu erlernen und einfach anzuwenden.
- Der TMS ist zeitintensiver, aber genauer in der Beurteilung von Krankheitsaktivität und Schädigung.
- MTX zeigt eine gute Wirksamkeit bei kutanen und extrakutanen Erscheinungen.
